# Peripheral ganglia in healthy rats as target structures for the evaluation of PSMA imaging agents

**DOI:** 10.1186/s12885-019-5841-8

**Published:** 2019-06-26

**Authors:** Heike Endepols, Agnieszka Morgenroth, Boris D. Zlatopolskiy, Philipp Krapf, Johannes Zischler, Raphael Richarz, Sergio Muñoz Vásquez, Bernd Neumaier, Felix M. Mottaghy

**Affiliations:** 1 Institute of Radiochemistry and Experimental Molecular Imaging, University of Cologne, Faculty of Medicine and University Hospital Cologne, 50937 Cologne, Germany; 2 Department of Nuclear Medicine, University of Cologne, Faculty of Medicine and University Hospital Cologne, 50937 Cologne, Germany; 3 Forschungszentrum Jülich GmbH, Institute of Neuroscience and Medicine, Nuclear Chemistry (INM-5), Wilhelm-Johnen-Straße, 52428 Jülich, Germany; 4 Department of Nuclear Medicine, University Hospital, RWTH Aachen, Pauwelsstraße 30, Aachen, 52074 Germany; 5 Department of Radiology and Nuclear Medicine, Maastricht University Medical Center X, Maastricht, the Netherlands

**Keywords:** Prostate carcinoma, Radiofluorination, Positron emission tomography, Imaging, Preclinical model

## Abstract

**Background:**

The recent implementation of PET with prostate specific membrane antigen (PSMA)-specific radiotracers into the clinical practice has resulted in the significant improvement of accuracy in the detection of prostate carcinoma (PCa). PSMA-expression in ganglia has been regarded as an important pitfall in prostate carcinoma-PET diagnostics but has not found any practical use for diagnosis or therapy.

**Methods:**

We explored this phenomenon and demonstrated the applicability of peripheral ganglia in healthy rats as surrogates for small PSMA positive lesions for the preclinical evaluation of diagnostic PCa PET probes. Healthy rats were measured with PET/CT using the tracers [^18^F]DCFPyL, [Al^18^F]PSMA-11 and [^68^Ga]PSMA-11. Sections of ganglia were stained with an anti-PSMA antibody. [^18^F]DCFPyL uptake in ganglia was compared to that in LNCaP tumor xenografts in mice.

**Results:**

Whereas [^18^F]DCFPyL and [^68^Ga]PSMA-11 were stable in vivo and accumulated in peripheral ganglia, [Al^18^F]PSMA-11 suffered from fast in vivo deflourination resulting in high bone uptake. Ganglionic PSMA expression was confirmed by immunohistochemistry. [^18^F]DCFPyL uptake and signal-to-noise ratio in the superior cervical ganglion was not significantly different from LNCaP xenografts.

**Conclusions:**

Our results demonstrated the non-inferiority of the novel model compared to conventionally used tumor xenografts in immune compromised rodents with regard to reproducibility and stability of the PSMA signal. Furthermore, the model involves less expense and efforts while it is permanently available and avoids tumor-growth associated animal morbidity and distress. To the best of our knowledge, this is the first tumor-free model suitable for the in vivo evaluation of tumor imaging agents.

## Background

Prostate-specific membrane antigen (PSMA) is a membrane bound type II zinc metallopeptidase which hydrolyzes *C*-terminal glutamate residues of small peptides in the extracellular space. The natural substrates of PSMA are the neurotransmitter *N*-acetylaspartylglutamate (NAAG) [1] and polyglutamate folate (pteroylpolyglutamate) [2]. PSMA expression is strongly upregulated in prostate carcinoma (PCa) and correlates with tumor malignancy (reviewed in [3]) making PSMA an attractive target for PCa diagnosis and treatment. In tumors, PSMA is supposed to be involved in folate uptake [4] as well as in laminin proteolysis which favor angiogenesis and re-endothelialization [5, 6]. The released glutamate binds to tumor cell metabotropic glutamate receptors stimulating tumor growth via phosphoinositide 3-kinase-related signaling pathways [7]. In recent years, PET or SPECT tracers for PSMA imaging have become important tools for PCa diagnostics [8–10]. In addition, radioligands labeled with therapeutic radionuclides like ^177^Lu and ^225^Ac have been successfully used for PSMA-targeted radiotherapy in prostate cancer [11, 12]. A suitable probe for molecular imaging or targeted radiotherapy should combine high specificity and long retention in target tissue with a rapid washout from non-target organs [13]. Usually, preclinical validation of novel radioligands for tumor imaging comprises in vitro studies of tracer uptake in target positive and negative cell lines as well as in vivo evaluation in appropriate animal models. The most common approaches employ tumor xenografts in immunodeficient animals [14]. While these models are generally well suited for assessment of target specificity and tumor binding kinetics, there are also some drawbacks. Since the blood vessels and stroma cells in human tumor xenografts are of murine origin, their growth is often irregular, and necrotic or cystic areas can be present already in small (8–10 mm) tumors. Poor blood supply can affect the penetration and distribution of imaging agents in tumors. Microbial or viral contamination of tumor cells could significantly affect not only survival and response of the immune deficient murine host but also properties of tumor tissues like immunogenicity, growth rate and metastatic potential [15]. Therefore, immunocompromised animals need to be housed and handled under specific pathogen-free conditions, which is laborious and expensive. For an initial in vivo screening of newly developed PSMA ligands, a facile alternative to xenograft models may be beneficial.

PSMA-specific uptake of PET tracers in human cervical, coeliac and sacral ganglia is well documented in the literature [16–18]. Similar to humans, rat ganglionic PSMA is expressed by satellite glial cells [19], which envelop the neuronal cell bodies of peripheral ganglia. Electron microscopic studies have shown that PSMA is mainly localized in the cell membrane of satellite cells [20]. PSMA/β-actin expression ratio is 1.5 for rat dorsal root ganglia [19], while it amounts to 6.8 for LNCaP prostate carcinoma cells [21]. PSMA in rat dorsal root ganglia shows an enzymatic activity of 9.9 ± 1.7 pmol NAAG/min/mg [20], while in the superior cervical ganglion PSMA activity (26.5 ± 0.2 pmol NAAG/min/mg) [20] is virtually equal to that of LNCaP cells (29.0 ± 2.6 pmol NAAG/min/mg) [22], which are broadly used for PCa xenograft models.

Having a size of only 1–2 mm, rat ganglia have the potential to mimic metastases in a very early stage. Rat PSMA comprises 752 amino acids (vs. 750 in humans), and is about 91% homologous to human PSMA. Importantly, all amino acid residues of the active site are essentially the same as those in the human homologue. The only exception is Ser^548^ in the rat instead of Gly^548^ in the human protein [23]. Not surprisingly, rat and human PSMA show comparable kinetic parameters for hydrolysis of NAAG and similar inhibition profiles [23]. We have already used peripheral ganglia of healthy rats for the preclinical screening of novel PSMA ligands [24]. This study resulted in discovery of a PSMA PET probe with favorable imaging properties which is already used in clinic [25, 26]. However, a thorough characterization of the model is still lacking. In this study we show that the ganglion model affords reproducible PET images with PSMA-specific PET tracers. PSMA-specificity of tracer binding is confirmed by immunohistochemistry and autoradiography. We examinated the applicability of this model using two widely used PSMA specific PET probes: [^68^Ga]PSMA-11 [27] and [^18^F]DCFPyL [28]. As an example of a PSMA-ligand with low in vivo stability we used PSMA-11 labeled with Al^18^F [29–31]. Finally, we compared [^18^F]DCFPyL PET-scans obtained in healthy rats with those from LNCaP tumor-bearing SCID mice.

## Methods

The aim of this study was to demonstrate the applicability of peripheral ganglia in healthy rats as surrogates for small PSMA positive lesions for the preclinical evaluation of PCa PET probes. Healthy rats were measured with small animal PET/CT using the tracers [^18^F]DCFPyL, [Al^18^F]PSMA-11 and [^68^Ga]PSMA-11. Anti-PSMA immunohistochemistry was used to confirm ganglionic PSMA expression. Rat superior cervical ganglia and mouse LNCaP tumor xenografts were compared with respect to [^18^F]DCFPyL uptake and signal-to-noise ratio.

### Production of [^18^F]fluoride

[^18^F]Fluoride was produced by the ^18^O(p,n)^18^F reaction by bombardment of 98% enriched [^18^O]water with 16.5 MeV protons at the MC16 cyclotron of the Max Planck Institute of Neurological Research in Cologne (Scanditronix, Uppsala, Sweden).

### Synthesis of tracers

[^18^F]DCFPyL was synthesized according to the modified procedure of Neumaier et al. (WO2016030329A1) using the “minimalist” protocol [32] and [Al^18^F]PSMA-11 according to Boschi et al. [30]. [^68^Ga]PSMA-11 was produced according to the method reported previously [33, 34].

Synthesis of 2,3,5,6-tetrafluorophenyl 6-[^18^F]fluoropyridine-3-carboxylate ([^18^F]FPy-TFP): Aqueous [^18^F]fluoride (0.05–50 GBq) was loaded onto a Sep-Pak Accell Plus QMA carbonate plus light cartridge (Waters GmbH, Eschborn, Germany) preconditioned with H_2_O (1 mL). The resin was washed with anhydrous EtOH (3 mL) and [^18^F]fluoride was eluted into the reaction vessel with a solution of [^18^F]FPy-TFP-precursor, *N*,*N*,*N*-trimethyl-5-[(2,3,5,6-tetrafluorophenoxy)-carbonyl]pyridine-2-aminium trifluoromethanesulfonate (10 mg, 21 μmol), in anhydrous EtOH (200 μL). Thereafter, the resin was washed into the reaction vessel with anhydrous MeCN/*t*BuOH 1:4 (2 mL). The mixture was stirred at 45 °C for 15 min. After cooling to ambient temperature, the reaction mixture was diluted with water (20 mL) and loaded onto a polymer RP cartridge (the cartridge was preconditioned with 2 mL EtOH followed by 30 mL H_2_O). The cartridge was washed with water (10 mL) and [^18^F]FPy-TFP (up to 25 GBq, radiochemical yield (RCY): 40–75% decay-corrected to EOB) was eluted with EtOH (500 μL). The radiochemical and chemical purities after SPE purification were > 98% determined by HPLC [eluent: 50% MeCN; flow rate: 1.5 mL/min; column: Chromolith® SpeedROD RP-18e column (Merck, Darmstadt Germany), 50 × 4.6 mm; retention time: 2.0 min].

Synthesis of [^18^F]DCFPyL: To a solution of {[(*S*)-5-amino-1-carboxypentyl]carbamoyl}-(*S*)-glutamic acid (Lys-CO-Glu, 2.5 mg, 7.8 mmol) in 0.19 m Et_4_NHCO_3_ in anhydrous EtOH (160 μL) [^18^F]FPy-TFP (1–10 GBq) in EtOH (500 mL) was added and the mixture was allowed to stir at 45 °C for 3–5 min. The mixture was quenched with H_2_O (2 mL) and purified by preparative HPLC (eluent: saline (0.9% NaCl) with 10% EtOH); flow rate: 8 mL/min, column: Synergi 4 μm Hydro-RP 80 Å 100 × 21.2 mm; retention time: 7.0 min). Finally, the purified product was passed through a sterile 0.2 μm filter. [^18^F]DCFPyL was prepared in 75–90% RCY. The molar activity amounted to 80–138 GBq/μmol (*n* = 35). Analytical HPLC conditions were as follows: eluent: 10% EtOH in aq. H_3_PO_4_ (pH 2) for 5 min, then 50% EtOH in aq. H_3_PO_4_ (pH 2) for 2 min; flow rate: 1.5 mL/min; column: Chromolith® SpeedROD RP-18e column (Merck, Darmstadt, Germany), 50 × 4.6 mm; retention times: [^18^F]DCFPyL 3.0 min and [^18^F]FPy-Tfp 5.8 min.

Synthesis of [Al^18^F]PSMA-11: Aqueous [^18^F]fluoride (0.05–50 GBq) was loaded onto a Sep-Pak Accell Plus QMA carbonate plus light cartridge (Waters GmbH, Eschborn, Germany) preconditioned with 0.5 m NaOAc (pH 4.5; 10 mL) followed by H_2_O (10 mL). The cartridge was washed with H_2_O (5 mL) and [^18^F]fluoride was eluted with 0.5 m NaOAc (pH 4.5; 0.5 mL). An aliquot of the resulting solution (200 μL) was added to a mixture of 6 μL 0.01 m AlCl_3_ in 0.05 m NaOAc (pH 4 and 50 μg Glu-NH-CO-NH-Lys(Ahx-HBED-CC) (PSMA-11) in 200 μL EtOH. The reaction mixture was allowed to stir at 100 °C for 5 min. After cooling to room temperature the crude product was passed through a HLB column, which was washed with NaOAc buffer (10 mL, pH = 4.3). The labeled product was eluted using 1:1 saline/EtOH (2 mL). [Al^18^F]PSMA-11 was prepared in 65–70% RCY. Analytical HPLC conditions were as follows: eluent: 5% for 3 min, then 50% for 7 min; flow rate: 3 mL/min; column: Chromolith® SpeedROD RP-18e column (Merck, Darmstadt, Germany), 50 × 4.6 mm; retention time: [Al^18^F]PSMA-11 5.7 min.

### Experimental animals

Animal experiments were carried out in accordance with the EU directive 2010/63/EU and the German Animal Welfare Act (TierSchG, 2006), and were approved by regional authorities (Ministry for Environment, Agriculture, Conservation and Consumer Protection of the State of North Rhine-Westphalia). Fourteen healthy male rats (6 Wistar, 7 Long Evans; Janvier Labs, Le Genest-Saint-Isle, France and Harlan Laboratories, Roßdorf, Germany; 380–635 g) and 5 male SCID mice (C.B-Igh-1b/IcrTac-Prkdcscid, Taconic, Hudson, USA; 18–19 g) were used. Rats were housed in pairs, mice in groups of 2–3 in individually ventilated cages (NexGen Ecoflo, Allentown Inc., Allentown, NJ, USA) under controlled ambient conditions (22 ± 1 °C and 55 ± 5% relative humidity). Food and water were available at all times. For tumor xenografts, a suspension of LNCaP cells (5 × 10^6^ in 150 μL matrigel) was injected subcutaneously in to the scruff of the neck between the shoulders of the mice. After 3 weeks, the mice developed subcutaneous tumors with volumes of about 1 cm^3^. The health status of all animals was monitored daily and was stable throughout the experiments. After completion of the measurements, the tumor-bearing mice were sacrificed by cervical dislocation. Apart from the one rat used for autoradiography, the rats were not sacrificed and remained in our animal facility.

### PET

A small animal PET/SPECT/CT scanner (Triumph II, Trifoil, USA) and a stand-alone small animal PET scanner (Focus 220, Siemens) were used. The tracer was injected during a short anesthesia (2% isoflurane in 30% oxygen/70% air) into the lateral tail vein ([^18^F]DCFPyL: 14–71 MBq, *n* = 6); [Al^18^F]PSMA-11: 25–54 MBq, *n* = 3; [^68^Ga]PSMA-11: 13–51 MBq, *n* = 3). Additionally, in three rats, [^18^F]DCFPyL was injected together with the PSMA inhibitor, 2-(phosphonomethyl)pentane-1,5-dioic acid (2-PMPA; 23 mg/kg).

Imaging started 60 min after tracer injection and ended 120 min after injection. Animals were fixed in an animal holder with anesthesia mask (2% isoflurane in 30% oxygen/70% air). Breathing rate and body temperature were monitored and held at approx. 60 breaths/min and 37 °C, respectively. Focus 220 emission scans were followed by an 8 min transmission scan with a rotating ^57^Co-point source, Triumph II emission scans by a 10 min CT scan (70 kV, 500 μA). The rats were sacrificed at the following day, and dorsal root ganglia were removed and fixed overnight in 4% paraformaldehyde in PBS (pH 7.4) for immunohistochemistry.

For mouse scans (*n* = 5), the Triumph II PET/SPECT/CT scanner was used, and 21–32 MBq of [^18^F]DCFPyL were injected i.v. Imaging procedure was as described above.

Following Fourier rebinning, data were reconstructed to single-frame summed images using an iterative OSEM3D/MAP procedure including attenuation and decay correction. Data analysis was performed with the help of the software VINCI 4.92 (Max Planck Institute for Metabolism Research, Cologne, Germany). Rat images were Gauss filtered (1 mm FWHM), mouse images remained unfiltered. SUV_BW_ was determined according to the following equation:

$$ {\mathrm{SUV}}_{\mathrm{BW}}=\mathrm{image}\ \mathrm{radioactivity}\ \left[\mathrm{Bq}/\mathrm{g}\right]\times \mathrm{body}\ \mathrm{weight}\ \left[\mathrm{g}\right]\times 100/\mathrm{injected}\ \mathrm{dose}\ \left[\mathrm{Bq}\right] $$

Elliptical volumes of interest (VOIs) were drawn to extract mean SUV_BW_ values for the rat trigeminal and superior cervical ganglia (9 mm^3^) and the mouse subcutaneous tumors (36–444 mm^3^, depending on tumor size). Background activity for calculation of signal-to-noise ratio was measured dorsal from the cervical vertebral column with a 390 mm^3^ VOI. For comparison of tracer uptake in the left and right superior cervical ganglion (SCG) of the same animal (*n* = 6 for [^18^F]DCFPyL, *n* = 3 for [^68^Ga]PSMA-11), the SCG with the higher mean SUV_BW_ was set to 100%, and the other SCG was proportioned accordingly (e.g. left SCG: 100%; right SCG: 98%). Side-specific mean values were calculated across animals.

### Autoradiography

Autoradiography of [^18^F]DCFPyL accumulation in rat dorsal root ganglia was performed with one healthy rat (635 g body weight). 70.5 MBq [^18^F]DCFPyL were injected during a short anesthesia (2% isoflurane in 30% oxygen/70% air) into the lateral tail vein. After 1 h of awake uptake the rat was sacrificed by cervical dislocation under anesthesia, and the cervical/thoracal spinal cord was extracted. Longitudinal sections of 50 μm thickness were cut on a cryostat and placed on a microscope slide. The sections were covered with scintillator foil and measured with a dFINE Betaimager (Biospace Lab, Paris, France) for 2 h.

### Immunohistochemistry

Consecutive formalin-fixed, paraffin-embedded longitudinal tissue sections (2 μm thick) of dorsal root ganglia were dewaxed in xylene and rehydrated through graded concentrations of ethanol to distilled water. Sections were then immersed in 10 mm sodium citrate buffer (pH 6.0) and processed for antigen retrieval in the microwave oven at 600 W for 5 min. For blocking of endogenous peroxidase activity, the sections were treated with 0.3% H_2_O_2_ solution for 15 min at ambient temperature. Unspecific antibody binding was inhibited by incubation in 3% BSA-TBST solution for 30 min at ambient temperature. For staining, sections were incubated with the anti-PSMA antibody (1:200 dilution in TBST, clone YPSMA-1, Abcam plc, Cambridge, UK) overnight at 4 °C. Subsequently, the sections were washed tree times with TBST and exposed for 60 min to peroxidase-linked anti-mouse immunoglobulin antibody (1:500 diluted in TBST, Cell Signaling Technology Europe B.V., Frankfurt-Main, Germany). Color development was performed using diaminobenzidine and sections were counterstained with haematoxylin/eosin.

### Statistical analysis

All statistical analyses were performed with Prism 6.0f for Mac OS X. Biodistribution of tracers was compared using One-way ANOVA followed by Dunnett’s multiple comparison test. Correlation between tumor tracer uptake and tumor size was assessed with the Pearson correlation test. Variances of tracer uptake in mouse tumor xenografts and rat SCG were compared with an F-Test for unequal variance.

## Results

### Biodistribution of PSMA specific tracers in healthy rats

**Fig. 1:**
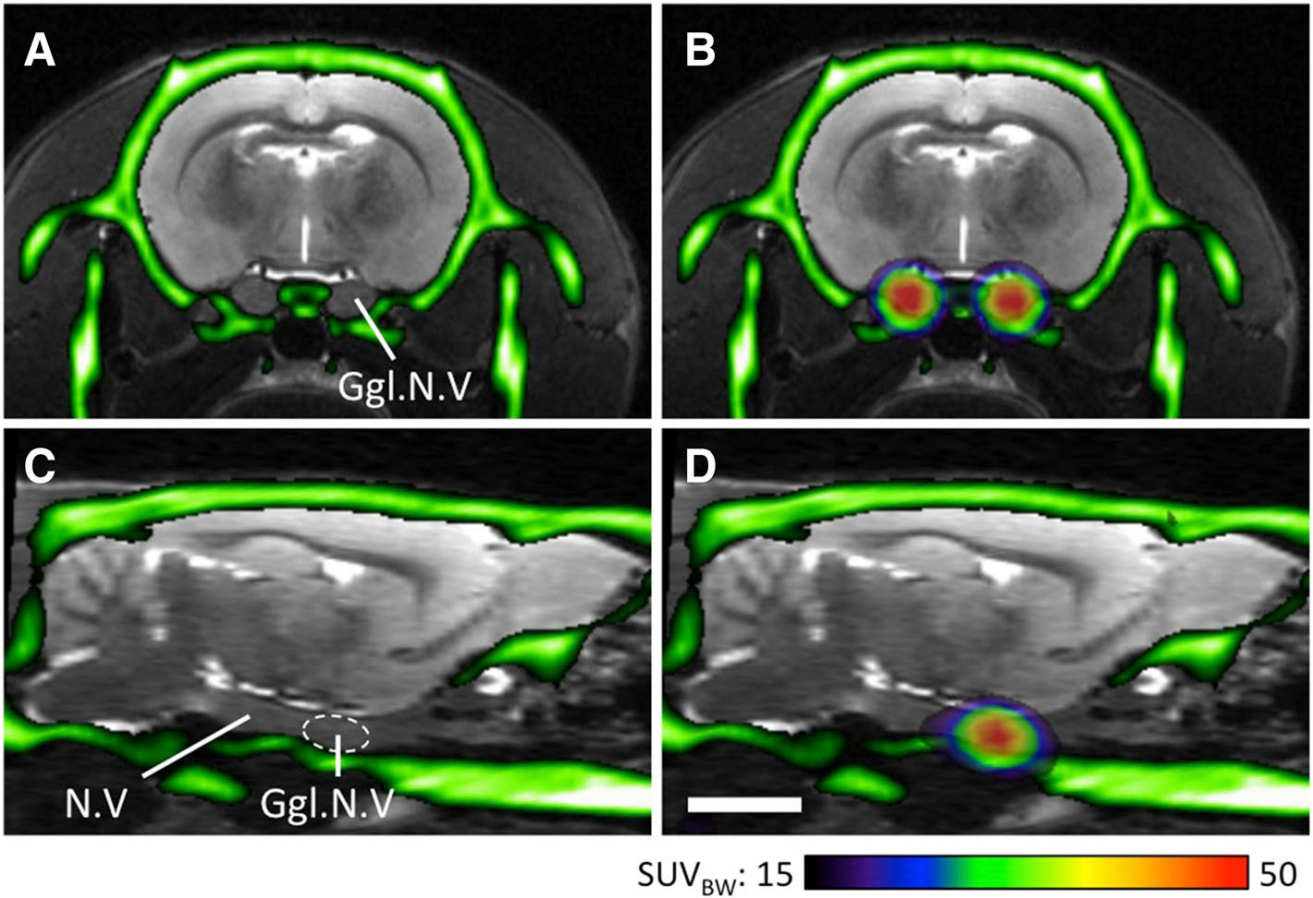
[^18^F]DCFPyl accumulation in the trigeminal ganglion. (**a**) + (**c**): Overlay of the CT image (green) and an MRI template (grey) in the transverse (**a**) and sagittal plane (**c**). (**b**) + (**d**): Overlay between CT (green), MRI (grey) and PET (colored). Abbreviations: Ggl.N.V: trigeminal ganglion; N.V: trigeminal nerve. Scale bar: 5 mm

**Fig. 2:**
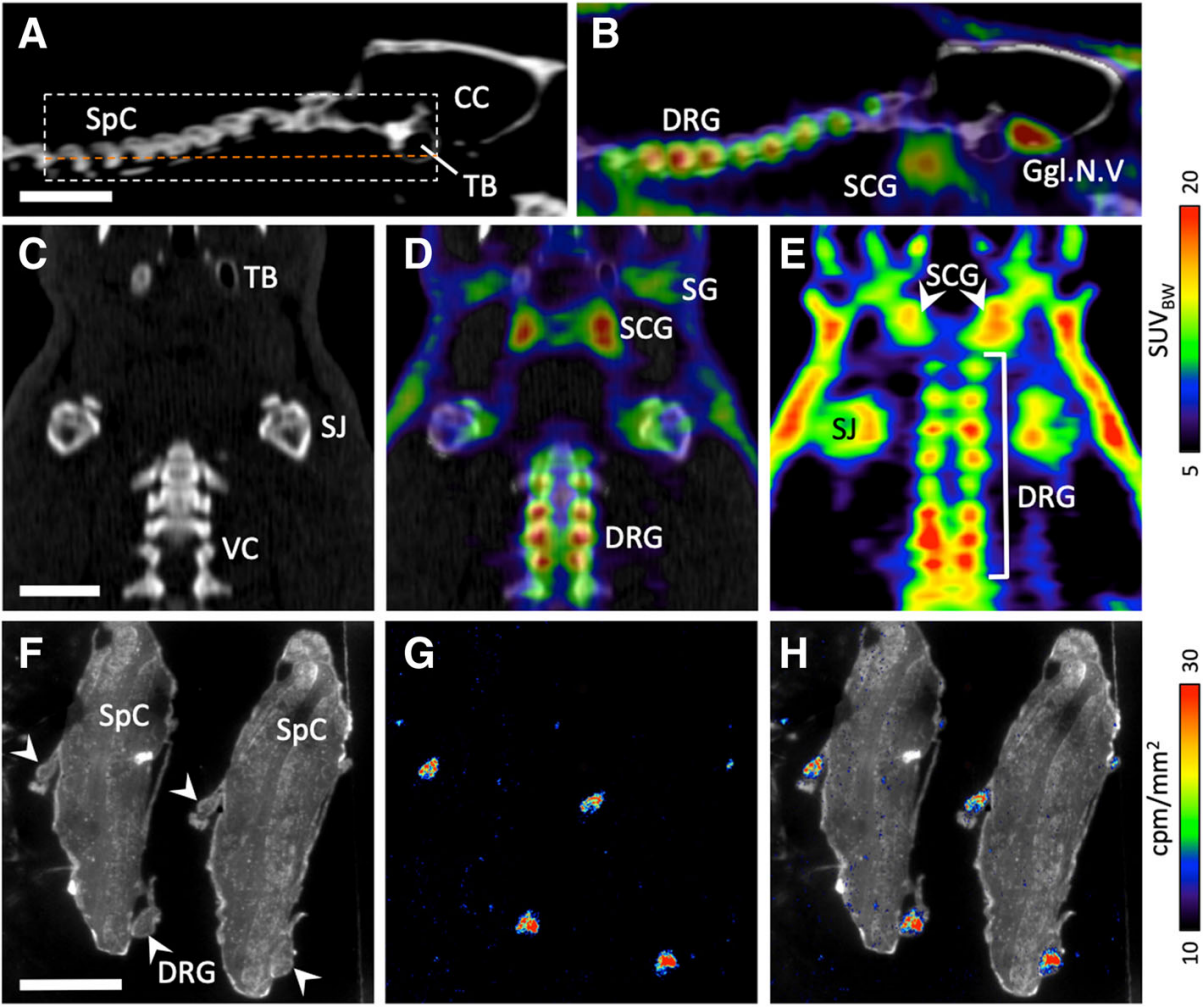
[^18^F]DCFPyL accumulation in dorsal root and autonomic ganglia. (**a**): Sagittal CT image of a rat skull and neck. (**b**): Overlay of PET and CT (sagittal). (**c**): Horizontal CT image of neck and shoulder region (along the orange dashed line in **a**). (**d**): Overlay of PET and CT (horizontal). (**e**): Sum of ten horizontal planes of the PET image, indicated by the box in (**a**). (**f**): Two longitudinal adjacent sections of a rat spinal cord. Dorsal root ganglia are indicated by arrowheads. (**g**): Autoradiographic image of spinal cord sections. (**h**): Overlay of microscopic and autoradiographic image. Abbreviations: CC: cranial cavity; DRG: dorsal root ganglia; Ggl.N.V.: ganglion of the trigeminal nerve; SCG: superior cervical ganglion; SG: salivary gland; SJ: shoulder joint; SpC: spinal cord; TB: tympanic bulla; VC: vertebral column. Scale bars: 1 cm in (**a**)–(**e**), 5 mm in (**f**)–(**h**)

**Fig. 3:**
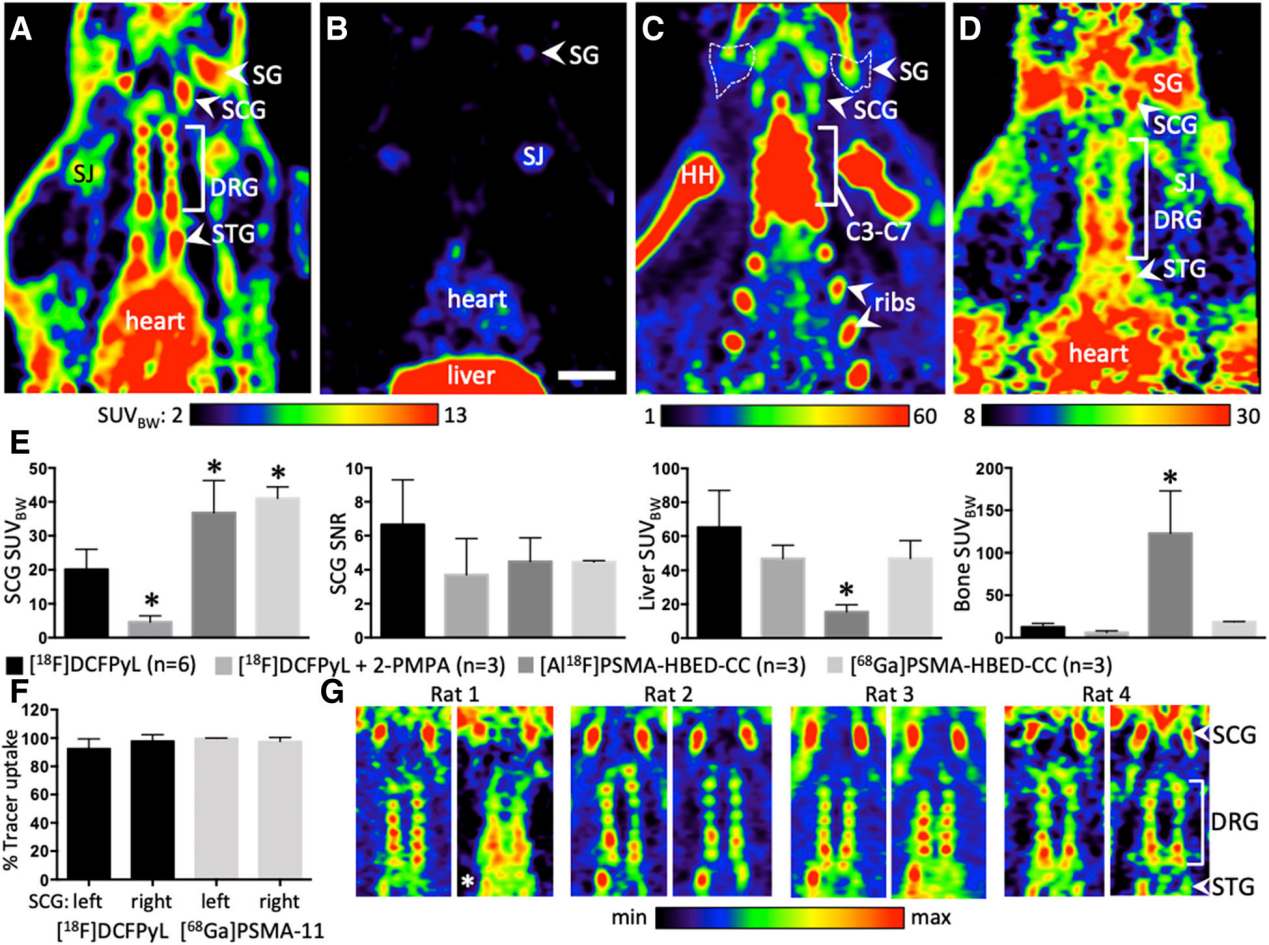
Cervical ganglia in healthy rats for validation of PSMA ligands. (**a**): Biodistribution of [^18^F]DCFPyL, horizontal plane. (**b**): Blocking experiment with [^18^F]DCFPyL + 2-PMPA (23 mg/kg). (**c**): Biodistribution of [Al^18^F]PSMA-11; high accumulation in bones  reflected substantial defluorination. (**d**): Biodistribution of [^68^Ga]PSMA-11. (**e**): Quantitative analysis of SCG tracer uptake, SCG signal-to-noise ratio, liver and bone (sternum) uptake. See also Table 1. (**f**): Similarity of tracer uptake in left and right SCG, shown for [^18^F]DCFPyL (*n* = 6, black bars) and [^68^Ga]PSMA-11 (*n* = 3, gray bars). (**g**): Four rats with two PSMA-PET measurements on different days, demonstrating stability of the ganglionic PSMA pattern. The second measurement in rat 1 was with [^68^Ga]PSMA-11, all others with ^18^F-labeled PSMA tracers (*). Abbreviations: C3–C7: cervical vertebrae 3–7; DRG: dorsal root ganglia; HH: humeral head (bone); SCG: superior cervical ganglion; SG: salivary gland; SJ: shoulder joint (cartilage); SNR: signal-to-noise ratio; STG: stellate ganglion. Scale bar: 1 cm

**Fig. 4:**
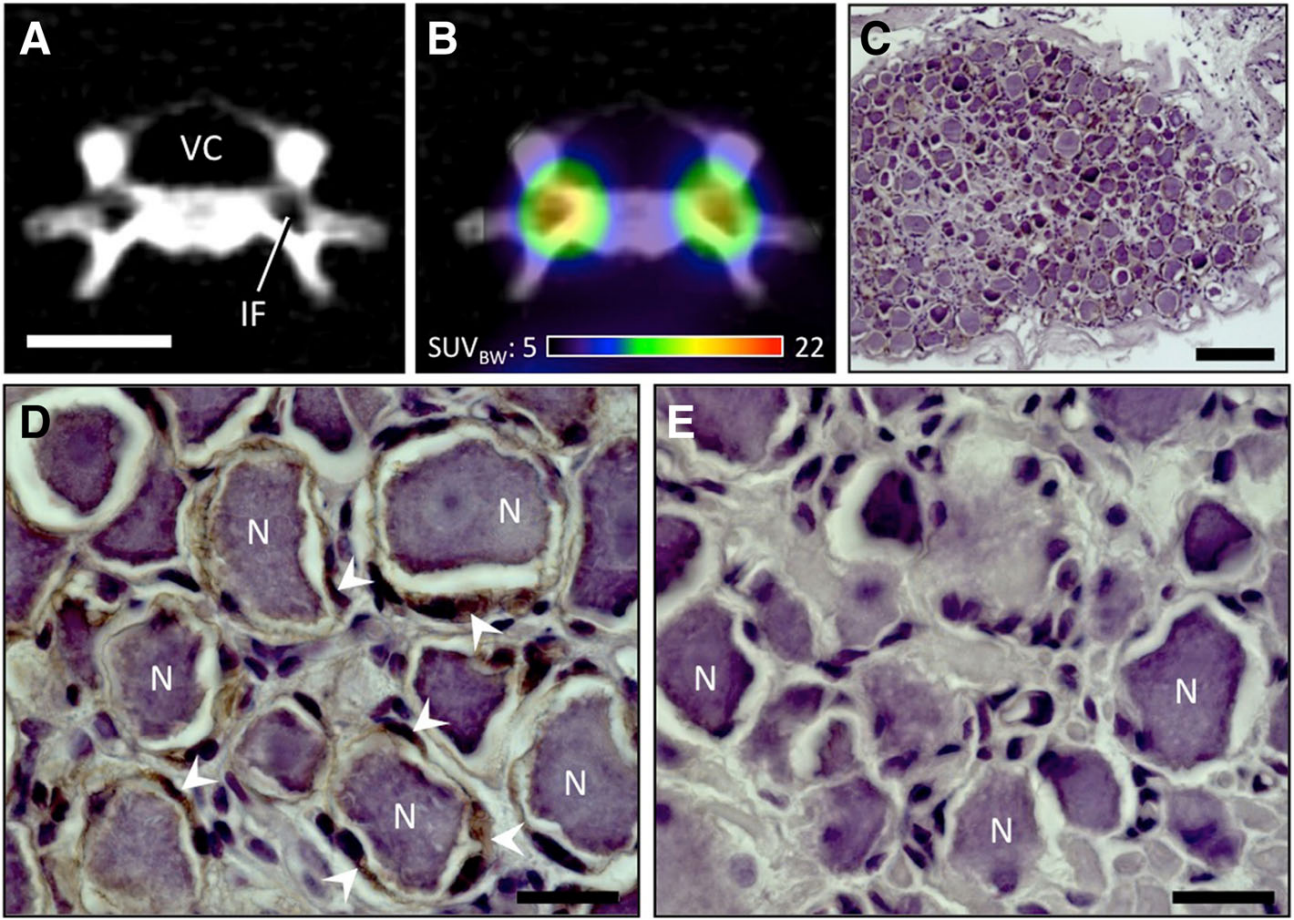
Localization of PSMA in dorsal root ganglia. (**a**): Transverse section through a CT image of the spinal column, between cervical vertebrae C6 and C7. (**b**): Overlay of PET and CT, showing tracer accumulation in the intervertebral foramina, where dorsal root ganglia are localized. (**c**): Overview of a dorsal root ganglion section, stained with H&E and anti-PSMA, corresponding to the radioactivity accumulation in the intervertebral foramen. (**d**): Zoomed fragment of (**c**), showing PSMA-immunostaining in satellite glial cells (arrowheads). (**e**): Control staining without primary antibody. Abbreviations: N: neuron; IF: intervertebral foramen; VC: vertebral canal. Scale bars: 5 mm in (**a**) + (**b**), 25 μm in (**c**) and 5 μm in (**d**) + (**e**)

In healthy rats, [^18^F]DCFPyL accumulated in peripheral ganglia with the highest uptake in the ganglion of the trigeminal nerve (37.9 ± 9.9 SUV_BW_, measured 60–120 min after injection; *n* = 6; Fig. 1). The eight focal accumulations of radioactivity detected in the cervical intervertebral foramina were assigned to the dorsal root ganglia (Figs. 2a-e, 3a, 4). Autoradiography confirmed that dorsal root ganglia, but not the spinal cord or the spinal nerves, accumulated [^18^F]DCFPyL (Fig. 2f-h). Radioactivity accumulation was also visible in the stellate ganglion, salivary glands and heart (Fig. 3a). The uptake of [^18^F]DCFPyL in the SCG was 20.2 ± 5.8 SUV_BW_ and the signal-to-noise ratio was 6.7 ± 2.6. Co-application with 2-PMPA strongly decreased radioactivity accumulation in the SCG (Fig. 3b; 4.6 ± 1.8 SUV_BW_) and also in other PSMA-positive tissues, whereas radioactivity enrichment in the liver was only slightly reduced ([^18^F]DCFPyL vs. [^18^F]DCFPyL + 2-PMPA: 65.3 ± 21.7 vs. 47.0 ± 7.7 SUV_BW_, see also Table 1).

**Table 1:** Accumulation of PSMA tracers in different tissues

	n	SCG, SUV_BW_	SNR	Liver, SUV_BW_	Bone, SUV_BW_
[^18^F]DCFPyL	6	20.2 ± 5.8	6.7 ± 2.6	65.3 ± 21.7	12.4 ± 4.3
[^18^F]DCFPyL + 2-PMPA	3	4.6 ± 1.8*	3.7 ± 2.1	47.0 ± 7.7	6.1 ± 1.9
[Al^18^F]PSMA-11	3	36.8 ± 9.5*	4.5 ± 1.4	15.5 ± 4.2*	122.8 ± 50.1*
[^68^Ga]PSMA-11	3	41.0 ± 3.4*	4.5 ± 0.1	47.0 ± 10.4	18.5 ± 0.61
ANOVA main effect		F (3, 11)=24.8 *p* < 0.0001	F (3, 11) =1.7 *p* = 0.2167	F (3, 11) = 6.7 *p* = 0.0079	F (3, 11) = 21.1 *p* < 0.0001

Abbreviations: *SCG*: superior cervical ganglion, *SNR* signal-to-noise ratio measured for SCG. *Significantly different from [^18^F]DCFPyL; *p* < 0.05 in Dunnett’s multiple comparison test

[Al^18^F]PSMA-11 suffered from substantial in vivo defluorination (Fig. 3c) reflected by high radioactivity uptake in bone (122.8 ± 50.1 SUV_BW_; F (3, 11) = 21.1; *p* < 0.0001; post-hoc *p* < 0.05). This bone uptake conceals tracer accumulation in the dorsal root ganglia which are embedded within the vertebrae. Nevertheless, tracer uptake in the SCG (36.8 ± 9.5 SUV_BW_; Fig. 3e) was significantly higher compared to [^18^F]DCFPyL (20.2 ± 5.8 SUV_BW_; F (3, 11) = 24.8; *p* < 0.0001; post-hoc *p* < 0.05), while signal-to-noise ratio was insignificantly lower (4.5 ± 1.4 vs. 6.7 ± 2.6; F (3, 11) = 1.7, *p* = 0.2167). Liver uptake of [Al^18^F]PSMA-11 was significantly lower (15.5 ± 4.2 SUV_BW_) compared to [^18^F]DCFPyL (65.3 ± 21.7 SUV_BW_; F (3, 11) = 6.7; *p* = 0.0079; post-hoc *p* < 0.05).

PET images obtained with [^68^Ga]PSMA-11 were more blurry with the overall background higher compared to that observed with the ^18^F-labeled tracers (Fig. 3d). While [^68^Ga]PSMA-11 uptake in the SCG (41.0 ± 3.4 SUV_BW_) was significantly higher compared to [^18^F]DCFPyL, signal-to-noise ratio (4.5 ± 0.1) was lower and similar to that of [Al^18^F]PSMA-11. Left and right SCG showed similar uptake of [^18^F]DCFPyL and [^68^Ga]PSMA-11 (Fig. 3f). When measured twice in the same animal, even with two different PSMA tracers, the individual shape of the SCG and dorsal root ganglia chain was clearly identifyable (Fig. 3g).

### Immunohistochemistry

Immunohistochemistry of dorsal root ganglia with a PSMA-specific antibody revealed that immunoreactivity was confined to the satellite glial cells, but absent in neurons (Fig. 4d). This finding confirms that the accumulation of radioactivity observed in the peripheral ganglia (Fig. 4b) reflects PSMA expression in the ganglionic satellite cells.

### Comparison with LNCaP xenografted mice

**Fig. 5:**
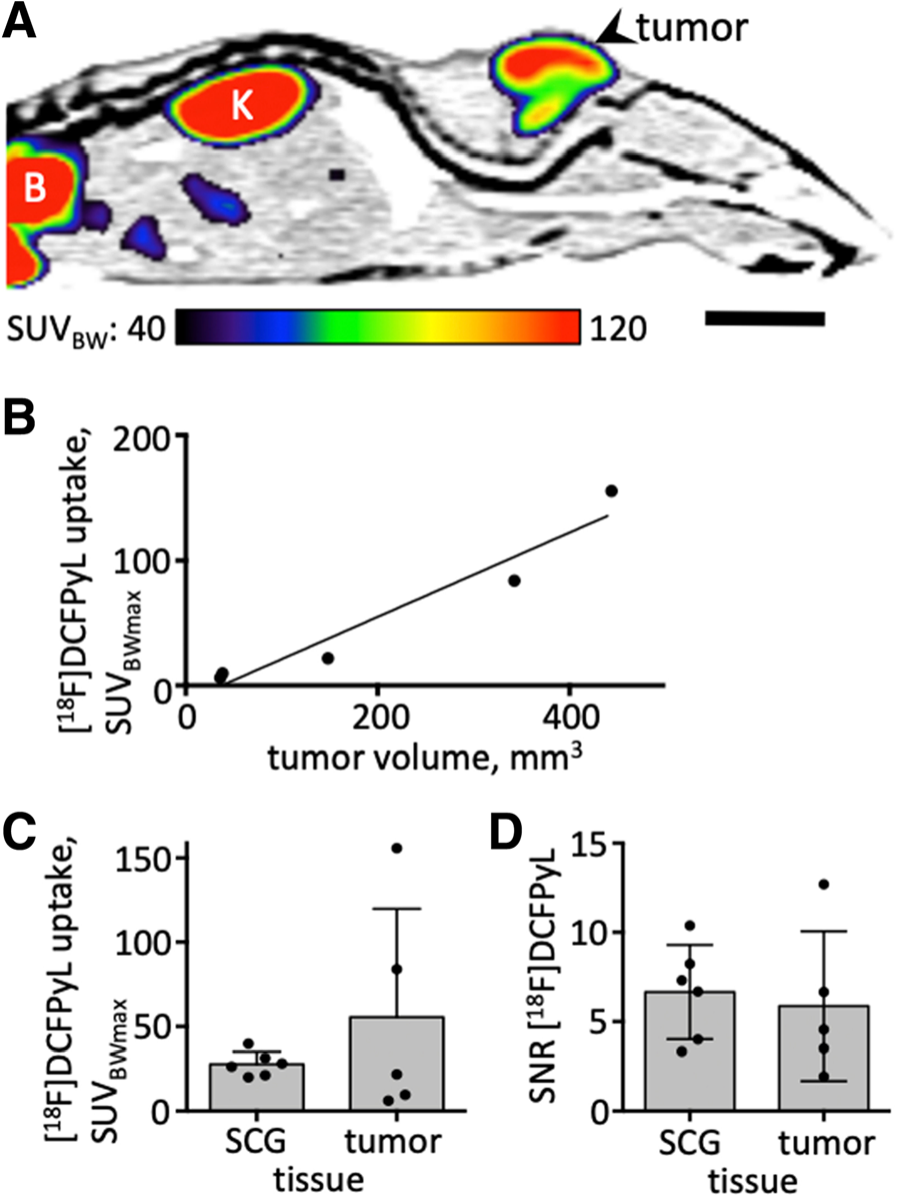
LNCaP PCa xenograft bearing mice. (**a**): [^18^F]DCFPyL, sagittal plane. (**b**): Maximal [^18^F]DCFPyL uptake (tumor voxel with highest intensity) plotted against tumor volume in five mice (R = 0.97, *p* = 0.0073). (**c**): Maximal [^18^F]DCFPyL uptake in the rat SCG compared to mouse tumor xenografts. Shown are mean ± standard deviation as well as individual data points. Variance was significantly higher in tumors compared to SCG (F (4, 5) = 78.4, *p* = 0.0002). (**d**): Signal-to-noise ratio measured with [^18^F]DCFPyL in rat SCG and mouse tumor xenografts. There was no significant difference between the two models. Abbreviations: B: bladder; K: kidney; SCG: superior cervical ganglion. Scale bar: 1 cm

In LNCaP xenografted mice, peripheral ganglia were also discernible, but were too small to provide delineated structures. Although LNCaP cells were inoculated on the same day, tumor sizes varied considerably (36–444 mm^3^). Tumor xenografts showed a heterogeneous uptake of [^18^F]DCFPyL (Fig. 5a). Therefore, we used the voxel with highest activity SUV_BWmax_ rather than the mean value of the whole tumor VOI. [^18^F]DCFPyL accumulation ranged from 6.2 SUV_BWmax_ to 155.8 SUV_BWmax_ depending on tumor size (R = 0.97; *p* = 0.0073; equation of regression line: y = 0.3377x–12.66; Fig. 5b). Signal-to-noise ratio was 5.8 ± 4.2. In small tumors (< 40 mm^3^) maximum [^18^F]DCFPyL uptake was below 10 SUV_BW_. According to the regression equation, tumors must reach a volume of 58 mm^3^ to achieve a maximum tracer uptake of 20.2 SUV_BW_, the average value measured for the rat SCG. The latter have a much smaller volume of approx. 9 mm^3^. Due to different tumor sizes, the variance of [^18^F]DCFPyL uptake in the mouse tumor xenografts was significantly higher compared to that in rat SCGs (F (4, 5) = 78.4, *p* = 0.0002; Fig. 5c).

## Discussion

Our results demonstrate that PSMA-targeted probes significantly accumulate in the rat peripheral ganglia due to specific binding to PSMA expressing satellite cells. Therefore, this structure might be used as a reference organ in the evaluation of PSMA ligands.

The rat superior cervical ganglia are easy-to-recognize tissues which fit in the axial field of view of typical μPET scanners together with the cervical dorsal root ganglia, heart and the frontal part of the liver. They are located far from any bone structures. Therefore, the sufficient spatial separation should exclude the possibility of significant “spillover” effects and allow quantification even in the case of tracer defluorination resulting in high accumulation of radioactivity in bone. Consequently, we choose SCG as a reference tissue for the exemplary comparative evaluation of PSMA ligands.

Our study showed significant differences between the tracers with regard to their image parameters. [^18^F]DCFPyL provided high quality visualization of the SCG with high signal-to-noise ratio. PSMA specificity of the uptake was confirmed by blocking experiments. Accumulation of [^68^Ga]PSMA-11 in the SCG was significantly higher compared to [^18^F]DCFPyL, whereas signal-to-noise ratio was comparable. The main difference between the two tracers was the lower perceived quality of images obtained with [^68^Ga]PSMA-11 presumably owing to longer positron range of ^68^Ga compared to that of ^18^F, as well as to scattered photons originating from high energy emission photons of ^68^Ga [35], but also higher background activity. Superior image quality of [^18^F]DCFPyL due to low beta energy of ^18^F was already described in patients [8, 36]. This effect was even more pronounced in small animals.

[Al^18^F]PSMA-11 combines the convenient half-life and low beta energy of ^18^F with the high PSMA affinity of [^68^Ga]PSMA-11 [29]. While the excellent properties of [Al^18^F]PSMA-11 with high SCG tracer accumulation and low liver retention (significantly higher and lower than those of [^18^F]DCFPyL, respectively) could be confirmed, we also observed high radioactivity uptake in bones attributed to extensive tracer defluorination. Whereas no substantial defluorination of [Al^18^F]PSMA-11 in vivo had been reported in earlier studies [29, 30], Ginglio et al. recently observed relatively rapid defluorination of the tracer in vitro in human serum with half-time of 2.5 h [31]. ^18^F-Fluoride avidly accumulates in PCa bone lesions [37], but also in healthy bones. This could lead to incorrect diagnosis and wrong therapy.

In the LNCaP tumor xenograft SCID mouse model, [^18^F]DCFPyL uptake linearly correlated with tumor size. This effect was described also for another PSMA ligand, [^64^Cu]CBT2G [38], and can presumably be attributed to poor vascularization [15] and, consequently, low blood supply of small tumor xenografts. Therefore, PSMA tracers should be evaluated in animals bearing xenografts of similar volumes to avoid inconsistent uptake data (Fig. 4c). Our results have shown that tumors require a size of 58 mm^3^ to match the [^18^F]DCFPyL uptake of the SCG. Although peripheral ganglia are small (< 10 mm^3^), they are rather densely vascularized [39], similar to metastases > 1 mm^3^ [40]. Therefore inter-individual variance of tracer accumulation in ganglia was low. The variance can be further reduced by application of the same healthy rats for the evaluation of several PSMA tracers. This is infeasible in tumor xenograft-bearing animals owing to the continuous tumor growth and tumor size-associated structural and metabolic tissue changes.

## Conclusions

While PSMA-expression in ganglia had been regarded as an important pitfall in prostate carcinoma-PET diagnostics [17] and had not found any practical use, we explored this phenomenon for the evaluation of PCa imaging agents in healthy rats.

We demonstrated the applicability of the peripheral ganglia of healthy rats as a non-invasive and inexpensive native model of small PCa lesions. Furthermore, our study revealed the non-inferiority of the novel model compared to conventionally used tumor xenografts in immune compromised rodents with regard to reproducibility and stability of the PSMA signal. The SCGs were always accessible for quantification of tracer uptake independently from tracer defluorination. The cervical dorsal root ganglia were also easily identifiable provided the radioligand was metabolically stable and defluorination did not occur. In contrast to the tumor xenograft model, where PSMA-specific uptake correlated linearly with tumor size, inter- and intra-individual variance of tracer accumulation in ganglia was low. Importantly, tumor burden-associated pain and adverse effects were avoided entirely. To the best of our knowledge, this is the first tumor-free model suitable for the in vivo evaluation of tumor imaging agents.

## Data Availability

The datasets used and/or analysed during the current study are available from the corresponding author on reasonable request.

## Abbreviations

[^18^F]DCFPyL: 2-(3-{1-carboxy-5-[(6-[(18)F]fluoro-pyridine-3-carbonyl)-amino]-pentyl}-ureido)-pentanedioic acid
2-PMPA: 2-(phosphonomethyl)pentane-1,5-dioic acid
BW: Body weight
CT: Computed tomography
EOB: End of bombardement
HPLC: High performance liquid chromatography
LNCaP: Lymph node carcinoma of the prostate
MRI: Magnetic resonance imaging
NAAG: *N*-acetylaspartylglutamate
PBS: Phosphate buffered saline
PCa: Prostate carcinoma
PET: Positron emission tomography
PSMA: Prostate-specific membrane antigen
RCY: Radiochemical yield
SCID: Severe combined immunodeficiency
SPECT: Single photon emission computed tomography
SUV: Standardized uptake value
VOI: Volume of interest
